# Genetic and lifestyle risk factors for MRI-defined brain infarcts in a population-based setting

**DOI:** 10.1212/WNL.0000000000006851

**Published:** 2019-01-29

**Authors:** Ganesh Chauhan, Hieab H.H. Adams, Claudia L. Satizabal, Joshua C. Bis, Alexander Teumer, Muralidharan Sargurupremraj, Edith Hofer, Stella Trompet, Saima Hilal, Albert Vernon Smith, Xueqiu Jian, Rainer Malik, Matthew Traylor, Sara L. Pulit, Philippe Amouyel, Bernard Mazoyer, Yi-Cheng Zhu, Sara Kaffashian, Sabrina Schilling, Gary W. Beecham, Thomas J. Montine, Gerard D. Schellenberg, Olafur Kjartansson, Vilmundur Guðnason, David S. Knopman, Michael E. Griswold, B. Gwen Windham, Rebecca F. Gottesman, Thomas H. Mosley, Reinhold Schmidt, Yasaman Saba, Helena Schmidt, Fumihiko Takeuchi, Shuhei Yamaguchi, Toru Nabika, Norihiro Kato, Kumar B. Rajan, Neelum T. Aggarwal, Philip L. De Jager, Denis A. Evans, Bruce M. Psaty, Jerome I. Rotter, Kenneth Rice, Oscar L. Lopez, Jiemin Liao, Christopher Chen, Ching-Yu Cheng, Tien Y. Wong, Mohammad K. Ikram, Sven J. van der Lee, Najaf Amin, Vincent Chouraki, Anita L. DeStefano, Hugo J. Aparicio, Jose R. Romero, Pauline Maillard, Charles DeCarli, Joanna M. Wardlaw, Maria del C. Valdés Hernández, Michelle Luciano, David Liewald, Ian J. Deary, John M. Starr, Mark E. Bastin, Susana Muñoz Maniega, P. Eline Slagboom, Marian Beekman, Joris Deelen, Hae-Won Uh, Robin Lemmens, Henry Brodaty, Margaret J. Wright, David Ames, Giorgio B. Boncoraglio, Jemma C. Hopewell, Ashley H. Beecham, Susan H. Blanton, Clinton B. Wright, Ralph L. Sacco, Wei Wen, Anbupalam Thalamuthu, Nicola J. Armstrong, Elizabeth Chong, Peter R. Schofield, John B. Kwok, Jeroen van der Grond, David J. Stott, Ian Ford, J. Wouter Jukema, Meike W. Vernooij, Albert Hofman, André G. Uitterlinden, Aad van der Lugt, Katharina Wittfeld, Hans J. Grabe, Norbert Hosten, Bettina von Sarnowski, Uwe Völker, Christopher Levi, Jordi Jimenez-Conde, Pankaj Sharma, Cathie L.M. Sudlow, Jonathan Rosand, Daniel Woo, John W. Cole, James F. Meschia, Agnieszka Slowik, Vincent Thijs, Arne Lindgren, Olle Melander, Raji P. Grewal, Tatjana Rundek, Kathy Rexrode, Peter M. Rothwell, Donna K. Arnett, Christina Jern, Julie A. Johnson, Oscar R. Benavente, Sylvia Wasssertheil-Smoller, Jin-Moo Lee, Quenna Wong, Braxton D. Mitchell, Stephen S. Rich, Patrick F. McArdle, Mirjam I. Geerlings, Yolanda van der Graaf, Paul I.W. de Bakker, Folkert W. Asselbergs, Velandai Srikanth, Russell Thomson, Rebekah McWhirter, Chris Moran, Michele Callisaya, Thanh Phan, Loes C.A. Rutten-Jacobs, Steve Bevan, Christophe Tzourio, Karen A. Mather, Perminder S. Sachdev, Cornelia M. van Duijn, Bradford B. Worrall, Martin Dichgans, Steven J. Kittner, Hugh S. Markus, Mohammad A. Ikram, Myriam Fornage, Lenore J. Launer, Sudha Seshadri, W.T. Longstreth, Stéphanie Debette

## Abstract

**Objective:**

To explore genetic and lifestyle risk factors of MRI-defined brain infarcts (BI) in large population-based cohorts.

**Methods:**

We performed meta-analyses of genome-wide association studies (GWAS) and examined associations of vascular risk factors and their genetic risk scores (GRS) with MRI-defined BI and a subset of BI, namely, small subcortical BI (SSBI), in 18 population-based cohorts (n = 20,949) from 5 ethnicities (3,726 with BI, 2,021 with SSBI). Top loci were followed up in 7 population-based cohorts (n = 6,862; 1,483 with BI, 630 with SBBI), and we tested associations with related phenotypes including ischemic stroke and pathologically defined BI.

**Results:**

The mean prevalence was 17.7% for BI and 10.5% for SSBI, steeply rising after age 65. Two loci showed genome-wide significant association with BI: FBN2, *p* = 1.77 × 10^−8^; and LINC00539/ZDHHC20, *p* = 5.82 × 10^−9^. Both have been associated with blood pressure (BP)–related phenotypes, but did not replicate in the smaller follow-up sample or show associations with related phenotypes. Age- and sex-adjusted associations with BI and SSBI were observed for BP traits (*p* value for BI, *p*_[BI]_ = 9.38 × 10^−25^; *p*_[SSBI]_ = 5.23 × 10^−14^ for hypertension), smoking (*p*_[BI]_ = 4.4 × 10^−10^; *p*_[SSBI]_ = 1.2 × 10^−4^), diabetes (*p*_[BI]_ = 1.7 × 10^−8^; *p*_[SSBI]_ = 2.8 × 10^−3^), previous cardiovascular disease (*p*_[BI]_ = 1.0 × 10^−18^; *p*_[SSBI]_ = 2.3 × 10^−7^), stroke (*p*_[BI]_ = 3.9 × 10^−69^; *p*_[SSBI]_ = 3.2 × 10^−24^), and MRI-defined white matter hyperintensity burden (*p*_[BI]_ = 1.43 × 10^−157^; *p*_[SSBI]_ = 3.16 × 10^−106^), but not with body mass index or cholesterol. GRS of BP traits were associated with BI and SSBI (*p* ≤ 0.0022), without indication of directional pleiotropy.

**Conclusion:**

In this multiethnic GWAS meta-analysis, including over 20,000 population-based participants, we identified genetic risk loci for BI requiring validation once additional large datasets become available. High BP, including genetically determined, was the most significant modifiable, causal risk factor for BI.

## Introduction

Brain infarcts (BI) detected on MRI are commonly seen in older persons, being described in 8%–28% of participants in population-based cohort studies.^1^ Most MRI-defined BI are covert, not being associated with overt, clinical stroke symptoms.^2,3^ Nonetheless, they cannot be considered silent or benign, as they are often associated with subtle neurologic symptoms and with increased risk of future stroke, cognitive decline, and in some studies dementia.^4,5^ Most MRI-defined BI are small subcortical BI (SSBI), believed to be primarily caused by small vessel disease (SVD).^6^

Mechanisms and predictors of BI and SSBI remain incompletely understood. No genetic risk variants for BI and SSBI have been consistently identified to date,^7–16^ and findings with vascular risk factors have been inconsistent.^1^ Partly reflecting this uncertainty, recommendations to direct clinicians on how to best manage covert MRI-defined BI are lacking.

To enhance understanding of risk factors for BI and SSBI, we first conducted a large meta-analysis of genome-wide association studies (GWAS) from 18 population-based studies, comprising 20,949 participants from 5 ethnic groups, using the 1000 Genomes reference panel (1000G), more than doubling the size of a prior GWAS.^16^ Second, we examined the association of vascular risk factors with BI and SSBI in this large sample, using both vascular risk factor measurements and their genetic risk scores (GRS).

## Methods

### Study design and samples

The meta-analyses included 18 prospective population-based cohorts participating in the Cohorts for Heart and Aging Research in Genomic Epidemiology (CHARGE) consortium (table e-1 and additional Methods e-1, doi.org/10.5061/dryad.hk07677). Although the cohorts contributing participants are longitudinal, this study is cross-sectional, based on the analysis of BI and SSBI at one timepoint in the subset of cohort participants with brain MRI. These cohorts comprised 5 ethnic groups and ancestries: European (n = 17,956), African (n = 1,834), Hispanic (n = 737), Malay (n = 215), and Chinese (n = 207). Some cohorts contributed to data for more than one ethnic group, resulting in a total of 23 datasets (tables e-1 to e-3, doi.org/10.5061/dryad.hk07677). Out of a total of 20,949 participants, 3,726 had MRI-defined BI. We did not exclude participants with a history of overt, clinically defined stroke prior to the MRI, except in 4 cohorts where patients with history of stroke were excluded by design. Three datasets did not contribute to the SSBI analysis either due to small numbers or absence of BI subtyping. Out of a total of 19,073 participants in the remaining 20 datasets, 3,533 had BI, of whom 2,021 (57.2%) had SSBI.

### Variable definitions

Detailed MRI scanning protocols, as well as BI and SSBI definitions, for each study are described in table e-4 (doi.org/10.5061/dryad.hk07677). All protocols comprised at least T1, T2, and proton density or fluid-attenuated inversion recovery (FLAIR) sequences. On MRI, BI were defined as an area of abnormal signal intensity lacking mass effect with a size ≥3–4 mm; in the white matter, they were required to be hypointense on T1-weighted images, approaching the hypointensity of CSF, to distinguish them from diffuse white matter lesions; and they were distinguished from dilated perivascular spaces based on their irregular shape, presence of a hyperintense rim in FLAIR, and absence of a typical vascular shape following the orientation of perforating vessels.^17^ SSBI corresponded to BI with a size <15–20 mm, located in the basal ganglia, the white matter, or the brainstem. Participants with large BI or BI located in the cerebral cortex or cerebellum were excluded from analyses of SSBI. We also measured burden of white matter hyperintensities (WMH), a quantitative MRI marker of SVD, corresponding to signal abnormalities of variable size in the white matter, appearing as hyperintensity on T2-weighted or FLAIR images, but without cavitation. Details of WMH measurements have been described previously.^18^

### Vascular risk factors

Vascular risk factor levels measured closest to brain MRI acquisition were used. Hypertension was defined as systolic blood pressure (SBP) ≥140 mm Hg or diastolic blood pressure (DBP) ≥90 mm Hg or use of one or more blood pressure (BP)–lowering medications. We defined pulse pressure (PP) as the difference between SBP and DBP and mean arterial pressure (MAP) as DBP + 1/3 × PP. Diabetes was defined as a previous diagnosis of diabetes, a fasting plasma glucose >7.0 mmol/L, or antidiabetic drug use. Fasting serum total cholesterol, high-density lipoprotein (HDL) cholesterol, and triglycerides were measured using enzymatic methods. Low-density lipoprotein (LDL) cholesterol was calculated using the Friedewald formula. Body mass index (BMI) was defined as the ratio of weight (kg) to the square of height (m). Active smoking was defined according to study-specific criteria. History of overt, clinically defined stroke and other cardiovascular events was based on ongoing surveillance prior to brain MRI acquisition in most studies since participant recruitment had started prior to the initial brain MRI. In studies that had brain MRI scanning at the initial visit, the history and examination at this visit were used to identify prior overt, clinically defined stroke. History of cardiovascular events included history of angina, myocardial infarction, cardiac bypass surgery, angioplasty, or peripheral vascular disease.

### Genotypes

All participating discovery cohorts had genome-wide genotypes imputed on the 1000G (phase 1, version 3).^19^ Genome-wide genotyping platforms, quality control measures, and imputation parameters used in each study are presented in tables e-5–e-7 (doi.org/10.5061/dryad.hk07677).

### Genome-wide association analyses with BI and small subcortical BI

For genome-wide association analyses with BI and SSBI, each study performed logistic regression under an additive genetic model after adjusting for age, sex, principal components of population stratification, and additional study-specific covariates, such as study site or family structure, as needed (additional Methods e-2, doi.org/10.5061/dryad.hk07677, for centralized quality control description). Our primary multiethnic GWAS meta-analysis was performed using MANTRA, based on a Bayesian framework.^20^ In secondary analyses, we also ran the multiethnic GWAS meta-analysis with 2 alternative methods (additional Methods e-2, doi.org/10.5061/dryad.hk07677): (1) using fixed effects inverse variance weighting with METAL^21,22^ and (2) using the random effects meta-analysis model implemented in METASOFT.^23^ During meta-analysis, genomic control correction was applied to the individual studies and ethnic-specific results to remove any residual inflation of association statistics. We did not observe any systematic inflation of association statistics (figure e-1, doi.org/10.5061/dryad.hk07677). Statistical measures from MANTRA, the primary meta-analysis method, were used to define genome-wide significance (Log_10_ of Bayesian factor [L_10_BF] > 6)^24^ and to choose single nucleotide polymorphisms (SNPs) for follow-up (L_10_BF > 4.5) in either the BI or SSBI meta-analysis. Details of functional annotation of top loci are provided in additional Methods e-3 (doi.org/10.5061/dryad.hk07677).

### Follow-up and extension

For follow-up and extension studies, genotypes imputed to the 1000G reference panel were available in most instances for in silico look-up of the selected risk variants. Three follow-up studies performed de novo genotyping of the top 6 loci (additional Methods e-1, doi.org/10.5061/dryad.hk07677). The lead variant (with lowest *p* value) was genotyped at each suggestive or genome-wide significant locus, and if not feasible, another variant in strong linkage disequilibrium (LD, *r*^2^ > 0.8) was genotyped. A *p* value <0.0083, correcting for 6 loci, was considered significant evidence for replication.

The follow-up sample, in which we sought to confirm associations observed in the discovery analysis, included 6,862 participants, of whom 1,483 had BI and 630 had SSBI, from 6 community-based studies of European origin and one of Japanese origin (table e-1, doi.org/10.5061/dryad.hk07677).

As an extension, to test whether genetic variants associated with MRI-defined BI or SSBI in the discovery analysis are also associated with correlated phenotypes, we first explored their association with ischemic stroke (IS) overall and the small vessel disease subtype (IS-SVD) when available in 4 collaborative studies (table e-1, doi.org/10.5061/dryad.hk07677). Second, we explored whether genetic variants associated with MRI-defined BI and SSBI were associated with neuropathologically defined BI based on 2,940 brain autopsies in participants without dementia from the Alzheimer's Disease Genetics Consortium (ADGC). Participants with large infarcts or lacunes (n = 857, 29%) were compared to participants without any infarcts or having only microscopic infarcts (n = 2,083).^25^

We calculated power of the follow-up and extension studies using Quanto V1.2.3 (biostats.usc.edu/software; table e-8 and figure e-2, doi.org/10.5061/dryad.hk07677).

### Association of vascular risk factors with BI and SSBI

Individual studies performed logistic regression to test for association of vascular risk factor measurements with presence or absence of at least one BI or SSBI. Analyses were performed with and without adjustments for age and sex. Analyses with BP or lipid traits as the main independent variable were additionally adjusted for treatment with disease-specific medications, and association analyses with fasting plasma glucose were limited to participants without type 2 diabetes. Except for WMH burden, the regression coefficients and standard errors for risk factors in the individual studies belonging to one ethnic group were combined using fixed-effects inverse variance-weighted meta-analysis and subsequently the betas and standard errors obtained in each ethnic group were combined using fixed-effects inverse variance-weighted meta-analysis, in the absence of heterogeneity (*p* < 1 × 10^−6^), to derive the multiethnic meta-analysis estimates. For WMH burden, the statistics were combined using the *Z* score–based sample size weighted meta-analysis as WMH burden was measured on different scales in participating studies.^18^

We then explored whether genetic variants previously shown in published GWAS to be associated with specific vascular risk factors were, in aggregate, also associated with BI and SSBI. This approach was selected to assess to what extent genetically determined vascular risk factor levels are associated with BI and SSBI and to provide evidence for a causal relation between a given vascular risk factor and risk of BI or SSBI, provided that Mendelian randomization assumptions are fulfilled.^26^ We combined known genetic risk variants for each individual vascular risk factor into a weighted GRS, using effect estimates from the largest published GWAS of that risk factor as weights. We then tested for association of these GRS with BI and SSBI using the inverse-variance weighting (IVW) method. Construction of the GRS, selection of variants for the GRS analysis, as well as effect estimates used as weights are detailed in additional Methods e-4 and tables e-9–e-12 (doi.org/10.5061/dryad.hk07677). For significant GRS associations with BI or SSBI, we further conducted sensitivity analyses using the MR-Egger method implemented as an R package (TwoSampleMR),^27^ which unlike the IVW method estimates the intercept term as part of the analysis. An intercept term significantly differing from zero suggests the presence of directional (unbalanced) pleiotropy, meaning that the pleiotropic effects of genetic variants are not balanced about the null.^27^ We used a conservative significance threshold of *p* < 0.05 for the intercept.

After Bonferroni correction for 12 independent vascular phenotypes tested for association with BI and SSBI, *p* < 0.0042 was considered significant for associations with vascular risk factor measurements or GRS. The number of independent vascular phenotypes, taking into account correlation between the phenotypes considered, was estimated based on individual level data from the 3C-Dijon study using the online tool matSpDlite (neurogenetics.qimrberghofer.edu.au/matSpDlite/).

### Standard protocol approvals, registrations, and patient consents

Institutional review boards approved all of these studies, and all participants provided informed consent.

### Data availability

Summary statistics of the top SNPs are available from Dryad for both BI and SSBI. Other data that support the findings of this study are available from the corresponding authors upon reasonable request.

## Results

**Figure 1:**
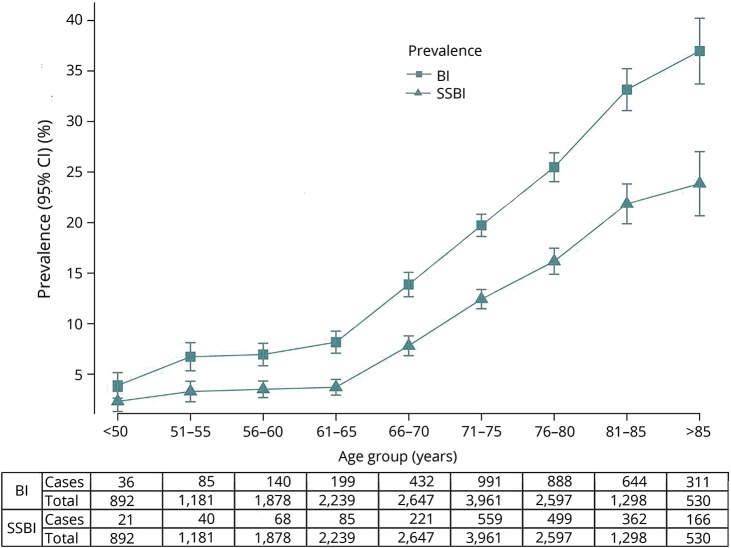
Prevalence of MRI-defined brain infarcts (BI) and small subcortical brain infarcts (SSBI) by different age groups CI = confidence interval.

In this large population-based dataset comprising 18 cohort studies, the frequency of MRI-defined BI ranged from 4% to 38% in participating cohorts (table e-1, doi.org/10.5061/dryad.hk07677). A description of demographic characteristics in all participants with BI (n = 3,726), with SSBI (n = 2,021), and without BI (n = 17,223) is provided in tables e-2 and e-3 (doi.org/10.5061/dryad.hk07677) for individual studies. Participants with BI and SSBI were on average 6 years older and more often men compared to those without BI. In age-stratified analyses, the prevalence of BI and SSBI increased with age, most prominently beyond age 65, after which a 25.8% (range 13.9%–37.0%) increment in BI prevalence was observed compared to participants younger than 65 years (figure 1). Overall, the prevalence of BI ranged from less than 5% before age 50 to over 30% beyond age 80, with similar findings when we analyzed men and women separately (figures e-3 and e-4, doi.org/10.5061/dryad.hk07677). Only 11% of those with BI and 9% of those with SSBI had a history of stroke (12.5% and 9.8% when removing cohorts that excluded participants with history of stroke by design); hence, the vast majority of MRI-defined BI were covert.

Genome-wide association plots for GWAS of BI and SSBI are displayed in figures e-5 and e-6 (doi.org/10.5061/dryad.hk07677). Two loci were associated with risk of BI at genome-wide significant level (L_10_BF > 6): rs39938 in *FBN2* (chr5q23) and rs12583648 in *LINC00539* and near *ZDHHC20* (chr13q12). In addition, 2 SNPs were associated with BI at a suggestive level of significance (L_10_BF > 4.5): rs12373108 near *CALB2/ZNF23* (chr16q22) and rs74587705 in *SV2B* (chr15q26) (table 1). No genome-wide significant association was observed for SSBI, but 2 loci reached the threshold for suggestive association (L_10_BF > 4.5): rs9371194 in *PLEKHG1* (chr6q25) and rs75889566 in *FRMD1* (chr6q27, table 1). These 6 loci were taken forward for the follow-up stage (table 2). For all SNPs reaching Log_10_BF > 4.5 in the discovery stage, association statistics are shown in table e-13 and figure e-7 (doi.org/10.5061/dryad.hk07677).

**Table 1:**
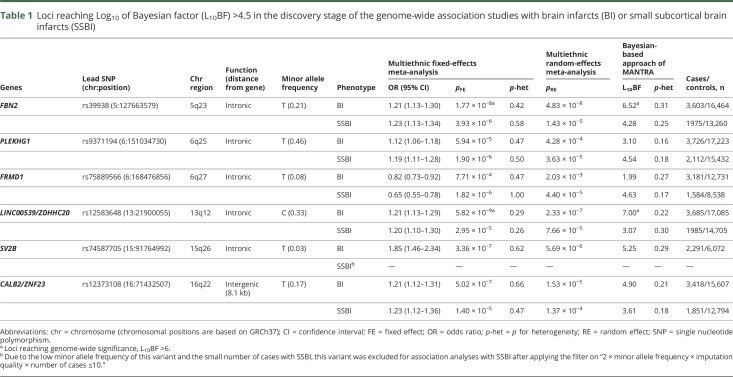
Loci reaching Log_10_ of Bayesian factor (L_10_BF) >4.5 in the discovery stage of the genome-wide association studies with brain infarcts (BI) or small subcortical brain infarcts (SSBI)

**Table 2:**
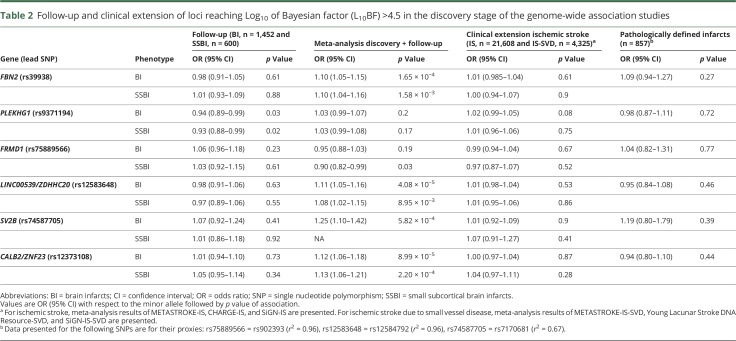
Follow-up and clinical extension of loci reaching Log_10_ of Bayesian factor (L_10_BF) >4.5 in the discovery stage of the genome-wide association studies

In the substantially smaller population-based follow-up studies, we could not replicate the 2 genome-wide significant or the 4 suggestive loci associated with BI or SSBI (table 2). Of the 6 loci that we followed up, we had limited power for 2 of the loci for BI (52%) and 4 of the loci for SSBI (50%–58%) (table e-8, doi.org/10.5061/dryad.hk07677). Power estimates in the follow-up study are even lower when accounting for the winner's curse phenomenon, which leads to inflated effect estimates in the discovery cohort.^28^ One suggestive locus for SSBI (*PLEKHG1*) showed nominal association with BI and SSBI in the follow-up studies (*p*_BI_ = 0.03 and *p*_SSBI_ = 0.02), but in the opposite direction (table 2).

Likewise, none of the genome-wide significant or suggestive loci for BI and SSBI showed association with IS (overall or IS-SVD) or pathologically defined BI in the extension studies after correcting for multiple testing (table 2 and table e-14, doi.org/10.5061/dryad.hk07677). Whereas the sample size for overall IS and IS-SVD was relatively large, it was limited for pathologically defined BI, and power was insufficient for 4 of the loci (25%–70%) (table e-8, doi.org/10.5061/dryad.hk07677).

Associations of vascular risk factors with risk of BI or SSBI adjusted for age and sex are presented in table 3 (for unadjusted results, see table e-15, doi.org/10.5061/dryad.hk07677). Both BI and SSBI were significantly associated with all BP indices, the lowest *p* value being observed for SBP and MAP. Smoking and diabetes were also associated with both BI and SSBI. Triglycerides were significantly associated with BI only. We did not observe significant associations with levels of HDL cholesterol, LDL cholesterol, BMI, or fasting plasma glucose in nondiabetic participants. Both BI and SSBI were associated with history of cardiovascular disease and history of stroke. The most significant association by far was observed with WMH burden on brain MRI, both for BI and SSBI. As hypertension is an important risk factor for WMH as well, we additionally adjusted the regression model for hypertension to rule out a confounding effect by this variable; however, the association became even more significant (*p* = 5.71 × 10^−172^ for BI and *p* = 4.47 × 10^−114^ for SSBI) (table e-16, doi.org/10.5061/dryad.hk07677). No significant heterogeneity was seen for these associations across participating studies.

**Table 3:**
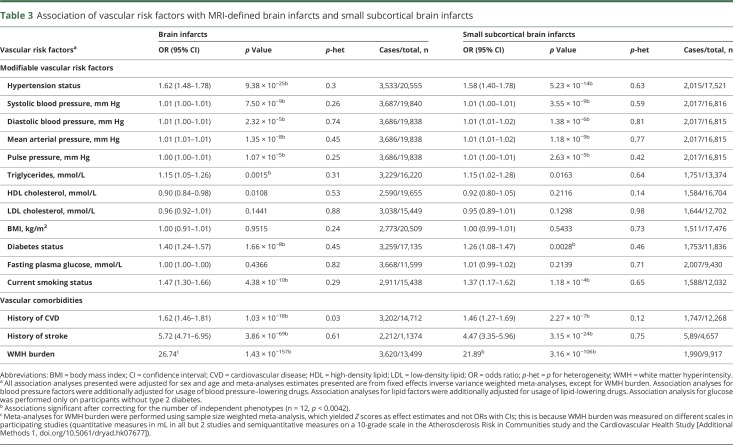
Association of vascular risk factors with MRI-defined brain infarcts and small subcortical brain infarcts

When exploring the relation of weighted genetic risk scores for vascular risk factors with BI and SSBI, we found that GRS for SBP and MAP were significantly associated with increased risk of BI and SSBI after correction for multiple testing (table 4). In sensitivity analyses using MR-Egger regression, evidence for directional pleiotropy was lacking for these associations between SBP or MAP GRS and BI or SSBI (*p* intercept >0.36). GRS for DBP, BMI, coronary artery disease, WMH burden, and IS were nominally associated with BI (*p* < 0.05, table 4), but these associations did not survive correction for multiple testing.

**Table 4:**
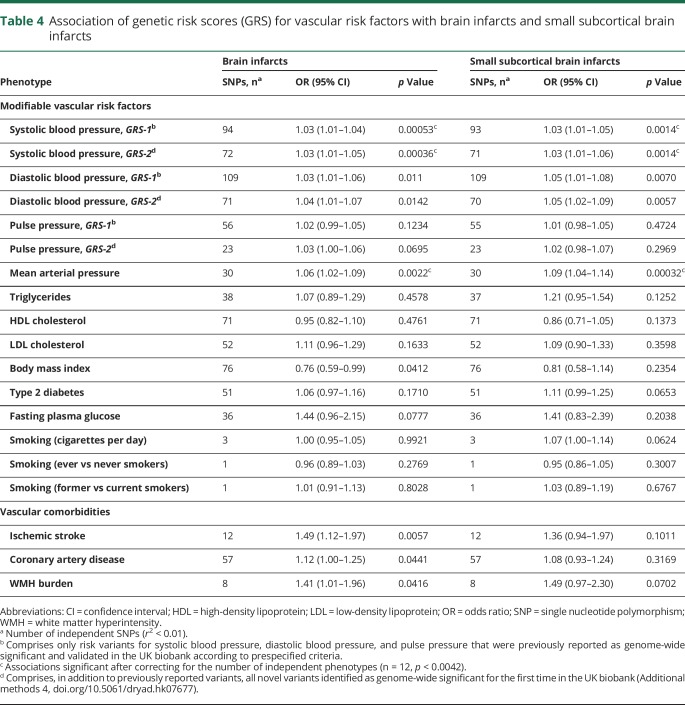
Association of genetic risk scores (GRS) for vascular risk factors with brain infarcts and small subcortical brain infarcts

## Discussion

This multiethnic meta-analysis comprising over 20,000 community participants provides noteworthy insight into risk factors for MRI-defined brain infarcts. The described BI distributions across different age groups and by sex may also serve as a reference for comparison with BI and SSBI frequency in other settings. Of note, about 90% of BI were covert, not being associated with a history of stroke. In this multiethnic GWAS of BI and SSBI, we identified 2 genome-wide significant risk loci for BI, *FBN2* on chr5q23 and *LINC00539/ZDHHC20* on chr13q12, although these could not be replicated in a smaller follow-up sample. We further describe the association of MRI-defined BI with vascular risk factors, combining the vast majority of population-based cohort studies with BI and SSBI measurements available. We find high BP, both phenotypically expressed high BP and genetically determined risk for high BP, to be the most significant modifiable risk factor for BI. No association with cholesterol levels or BMI was found.

To identify novel genetic risk loci for MRI-defined BI and SSBI, we have more than doubled the sample size compared to the previously published GWAS of MRI-defined BI,^16^ used imputed genotypes based on the 1000G reference panel to increase the marker coverage, and included samples from 5 ethnicities for a broader representation of individuals from different origins. Moreover, we studied both BI and SSBI, while only BI were analyzed in the previously published GWAS meta-analysis.^16^ Our inability to replicate the genome-wide significant and suggestive findings could reflect false-positive results but may also be explained by insufficient power in the follow-up stage (table e-8, doi.org/10.5061/dryad.hk07677). Further studies on larger samples with MRI-defined BI are required to confirm or refute these findings. Moreover, while we could not provide evidence for an association of genome-wide significant and suggestive risk loci for BI and SSBI with IS, IS-SVD, or pathologically defined BI, this inability could reflect differences in the biology underlying these phenotypes, as well as limited power in the extension studies.

The 2 loci that crossed the genome-wide significance threshold, while requiring confirmation in larger independent samples, do harbor plausible biological candidates. *Fibrillin2* (*FBN2*) encodes a protein that is part of the connective tissue microfibrils and elastic fiber assembly of the cell.^29^ Rare and common variants in *FBN2* have been associated with age-related macular degeneration.^30^ Recent studies have also implicated common variants in *FBN2* to be associated with SBP,^31^ although the variants differ (rs6595838-SBP and rs39938-BI, *r*^2^ = 0.017). The *LINC00539/ZDHHC20* locus was a suggestive hit in a GWAS of adverse metabolic response to hydrochlorothiazide, a drug commonly used to treat hypertension.^32^ The lead SNP in the region could also influence the expression of the long noncoding RNA *LINC00539* (table e-17, doi.org/10.5061/dryad.hk07677).

Our findings provide definitive evidence for a major and predominant association of increasing BP levels with increased risk of BI and SSBI.^1,33^ Beside significant associations with hypertension, a continuous association was observed for increasing levels of all BP measurements (SBP, DBP, PP, MAP), consistent with elevated BP being the major modifiable risk factor for BI, as is the case for overt, clinically defined IS.^34–36^ The importance and causal nature of the relation between high BP and risk of BI and SSBI is further supported by the significant association of BP genetic risk scores, for SBP and MAP, with increased risk of BI, especially SSBI, with no indication of directional pleiotropy using the MR-Egger approach.^27^

Previous publications on the association of BI and SSBI with vascular risk factors other than elevated BP were inconsistent.^1,33,37^ Our study provides evidence for a significant association of current smoking and diabetes with risk of BI and SSBI, while no association with BMI and cholesterol could be demonstrated, despite the very large sample size. These findings are consistent with epidemiologic data on IS.^35^ Interestingly, in contrast with cholesterol levels, a significant association of increasing triglyceride levels with BI risk was observed, although for SSBI the association did not withstand correction for multiple testing. Inconsistent results have been reported regarding association of triglycerides with overt, clinically defined IS,^38,39^ but the present results are in line with evidence of an association in older community-dwelling persons between high triglyceride levels and WMH burden, another MRI marker of SVD.^40^

As previously described, we show a significant association of WMH burden with BI and SSBI, reaching *p* < 10^−100^ in this study. Surprisingly, shared genetic variation among the top loci for WMH burden and BI was limited. While this observation could be due to lack of power, it could also suggest that WMH and BI share more environmental than genetic risk factors. A more comprehensive search for shared genetic variation between WMH burden and BI or SSBI at the genome-wide level using the LD score regression method^41^ could not be performed in the present study due to low variance in the BI GWAS, also hampering the calculation of BI heritability using the same method. Of note, based on estimates from previously published family-based studies, heritability for SSBI was described to be low at 29%, in contrast with a moderate to high heritability for WMH burden at 49%–80%.^42–44^ Hypertension is a major risk factor for WMH as well, and a BP GRS was also significantly associated with WMH burden in a prior study.^18^ However, the association of WMH burden with BI and SSBI was still significant after adjusting for hypertension status (table 3), or for SBP levels and BP-lowering treatment (table e-16, doi.org/10.5061/dryad.hk07677), suggesting that BP is not the only mediator of this association.

An important strength of the present study is that we have gathered nearly all large population-based studies with MRI-based identification of BI, genome-wide genotypes, and detailed vascular risk factor and comorbidity assessment, totaling over 20,000 participants covering 5 ethnic groups. Despite the unprecedented sample size, we were underpowered for the discovery of novel, robust genetic risk loci and even more so for the follow-up of genome-wide significant findings. Our ability to discover robust genetic risk variants may also have been hampered by the heterogeneity in BI and SSBI etiology, even though SVD is likely the predominant mechanism,^45^ and by some heterogeneity in the way BI and SSBI have been measured in participating studies. Finally, although the majority of participants had covert BI, 10% had a history of overt, clinically defined stroke, but including both covert and overt BI also enables a better representation of the spectrum of participants with MRI-defined BI in the general population. Whereas history of stroke was more common in participants with BI than those without, we do not believe that this inclusion has driven the associations we observed, given both the small number of participants with a stroke history and the significance level of the observed associations. Moreover, in this population-based setting, determining whether an MRI-defined BI could be attributed to the history of clinically defined stroke was not always possible.

In clinical practice, MRI-defined BI are commonly seen on brain MRI scans performed for various reasons in older persons. They have been shown to be powerful predictors of incident stroke and incident dementia.^1,4,46^ Hence BI represent an important marker for detection of high-risk individuals and initiation of preventive interventions. However, no randomized trials and no recommendations are currently available for the management of covert MRI-defined BI. The observational evidence is overwhelming for a strong causal relation between high BP and risk of BI and SSBI. A randomized trial will be needed to decide if persons with MRI-defined BI will benefit from more intensive BP-lowering strategies than is recommended currently for primary prevention.

This multiethnic, population-based study on 20,949 participants sheds important new light on susceptibility factors of MRI-defined brain infarcts, a marker of covert vascular brain injury commonly observed in older persons.

## Author affiliations

From the Bordeaux Population Health Research Center (G.C., M.S., S.K., S. Schilling, C.T., S.D.), INSERM U1219, Groupe d'Imagerie Neurofonctionnelle CNRS/CEAU5293 (B.M.), and University of Bordeaux (G.C., C.T., M.S., S.K., S. Schilling, S.D., B.M.), Department of Neurology, Bordeaux University Hospital (S.D.), Bordeaux, France; Departments of Epidemiology (H.H.H.A., M.W.V., A.H., M.A.I.), Radiology & Nuclear Medicine (H.H.H.A., M.W.V., A.v.d.L., M.A.I.), Internal Medicine (A.G.U.), and Neurology (M.A.I.), Erasmus MC, Rotterdam, the Netherlands; Department of Neurology (C.L.S., V.C., H.J.A., J.R.R., P.M., S. Seshadri), Boston University School of Medicine; Department of Biostatistics (A.L.D., S. Seshadri), Boston University School of Public Health; The National Heart, Lung, and Blood Institute's Framingham Heart Study (C.L.S., V.C., A.L.D., H.J.A., J.R.R., P.M., S. Seshadri), MA; Cardiovascular Health Research Unit, Department of Medicine (J.C.B., B.M.P.), and Departments of Epidemiology (B.M.P., W.T.L.), Health Services (B.M.P.), Biostatistics (K. Rice, Q.W.), Neurology (W.T.L.), and Pathology (T.J.M.), University of Washington, Seattle; Pathology (T.J.M.), Standford University, California; Institute for Community Medicine (A. Teumer), Department of Psychiatry and Psychotherapy (H.J.G.), Institute of Diagnostic Radiology and Neuroradiology (N.H.), and Department of Neurology (B.v.S.), University Medicine Greifswald, Germany; Clinical Division of Neurogeriatrics, Department of Neurology (E.H., R.S.), Institute for Medical Informatics, Statistics and Documentation (E.H.), and Gottfried Schatz Research Center (for Cell Signaling, Metabolism and Aging), Institute of Molecular Biology and Biochemistry (Y.S., H.S.), Medical University of Graz, Austria; Department of Cardiology (S.T., J.W.J.), Section of Gerontology and Geriatrics, Department of Internal Medicine (S.T.), Molecular Epidemiology (P.E.S., M.B., J.D.), Medical Statistics and Bioinformatics (H.-W.U.), and Department of Radiology (J.v.d.G.), Leiden University Medical Center, the Netherlands; Department of Pharmacology (S.H., C.C., M.K.I.) and Department of Ophthalmology, Yong Loo Lin School of Medicine (C.-Y.C.), National University of Singapore; Icelandic Heart Association (A.V.S., V.G.), Kópavogur, Iceland; Institute of Molecular Medicine (X.J., M.F.) and Human Genetics Center (M.F.), University of Texas Health Science Center at Houston; Institute for Stroke and Dementia Research (R.M., M.D.), Klinikum der Universität München, Ludwig-Maximilians-Universität, Munich, Germany; Clinical Neurosciences (M.T., L.C.A.R.-J., H.S.M.), University of Cambridge, UK; School of Life Sciences (S.B.), University of Lincoln, United Kingdom; German Center for Neurodegenerative Diseases (DZNE) (L.C.A.R.-J.), Population Health Sciences, Bonn, Germany; Department of Medical Genetics (S.L.P.), Department of Neurology, Brain Center Rudolf Magnus (M.K.I.), Department of Epidemiology, Julius Center for Health Sciences and Primary Care (M.I.G., Y.v.d.G.), Department of Genetics, Center for Molecular Medicine (P.I.W.d.B.), and Department of Cardiology, Division Heart & Lungs (F.W.A.), University Medical Center Utrecht, and Utrecht University, the Netherlands; Institut Pasteur de Lille (V.C., P.A.), Lille University, INSERM, Lille University Hospital, France; Department of Neurology (Y.-C.Z.), Peking Union Medical College Hospital, Beijing, China; John P. Hussman Institute for Human Genomics (G.W.B., A.H.B., S.H.B.), Department of Neurology (R.L.S., T.R.), Evelyn F. McKnight Brain Institute (R.L.S., T.R.), Department of Epidemiology and Public Health Sciences (R.L.S., T.R.), and Dr. John T. Macdonald Foundation Department of Human Genetics (R.L.S., S.H.B., A.H.B.), Miller School of Medicine, University of Miami, FL; Department of Pathology and Laboratory Medicine (G.D.S.), University of Pennsylvania School of Medicine, Philadelphia; Departments of Neurology & Radiology (O.K.), Landspitali National University Hospital; Faculty of Medicine (V.G.), University of Iceland, Reykjavik; Department of Neurology (D.S.K.), Mayo Clinic, Rochester, MN; Departments of Data Science (M.E.G.) and Medicine (B.G.W., T.H.M.), University of Mississippi Medical Center, Jackson; Department of Neurology (R.F.G.), Johns Hopkins University School of Medicine, Baltimore, MD; Department of Gene Diagnostics and Therapeutics (F.T., N.K.), Research Institute, National Center for Global Health and Medicine, Tokyo; The Third Department of Internal Medicine (S.Y.) and Department of Functional Pathology (T.N.), Shimane University School of Medicine, Japan; Rush University Medical Center (K.B.R., N.T.A., D.A.E.), Chicago, IL; Brigham and Women's Hospital (K. Rexrode); Center for Translational & Computational Neuroimmunology, Department of Neurology (P.L.D.J.), Columbia University Medical Center, New York, NY; Kaiser Permanente Washington Health Research Institute (B.M.P.), Seattle, WA; Institute for Translational Genomics and Population Sciences, Los Angeles Biomedical Research Institute, and Division of Genomic Outcomes, Department of Pediatrics (J.I.R.), Harbor-UCLA Medical Center, Torrance; Departments of Pediatrics, Medicine, and Human Genetics (J.I.R.), UCLA, Los Angeles, CA; Department of Neurology (O.L.L.), University of Pittsburgh, PA; Singapore Eye Research Institute (J.L.); Duke-NUS Graduate Medical School (C.-.YC., T.Y.W., M.K.I.), Singapore; Singapore Eye Research Institute (C.-.YC., T.Y.W., M.K.I.), Singapore National Eye Centre; Memory Aging & Cognition Centre (MACC), National University Health System (M.K.I.), Singapore; Genetic Epidemiology Unit, Department of Epidemiology and Biostatistics (S.J.v.d.L., N.A., C.M.v.D.), Erasmus MC University Medical Center, Rotterdam, the Netherlands; Department of Neurology (C.D.), University of California at Davis; Brain Research Imaging Centre (J.M.W., M.d.C.V.H., M.E.B., S.M.M.), Centre for Clinical Brain Sciences (J.M.W., M.d.C.V.H., M.E.B., C.L.M.S.), Edinburgh Dementia Research Centre (J.M.W., M.d.C.V.H.), Centre for Cognitive Ageing and Cognitive Epidemiology (J.M.W., M.d.C.V.H., M.L., D.L., I.J.D., M.E.B., J.M.S., S.M.M.), Alzheimer Scotland Dementia Research Centre (J.M.S.), and Institute of Genetics and Molecular Medicine (C.L.M.S.), University of Edinburgh, UK; Department of Neurosciences, Experimental Neurology and Leuven Research Institute for Neuroscience and Disease (LIND) (R.L.), KU Leuven–University of Leuven; Center for Brain & Disease Research (R.L.), VIB, Laboratory of Neurobiology; Department of Neurology (R.L.), University Hospitals Leuven, Belgium; Florey Institute of Neuroscience and Mental Health (V.T.), University of Melbourne, Australia; Centre for Healthy Brain Ageing, Psychiatry (H.B., W.W., A. Thalamuthu, N.J.A., E.C., K.A.M., P.S.S.), Dementia Centre for Research Collaboration (H.B.), and School of Medical Sciences (P.R.S., J.B.K.), University of New South Wales, Sydney; Mathematics & Statistics (N.J.A.), Murdoch University, Perth; Neuroscience Research Australia (K.A.M., P.R.S., A.Thalamuthu), Randwick; Brain and Mind Centre (J.B.K.), The University of Sydney, Camperdown; Queensland Brain Institute (M.J.W.), University of Queensland, Brisbane; National Ageing Research Institute (D.A.), Melbourne; Academic Unit for Psychiatry of Old Age (D.A.), University of Melbourne, Australia; Department of Cerebrovascular Diseases (G.B.B.), Fondazione IRCCS Istituto Neurologico “Carlo Besta,” Milan, Italy; CTSU, Nuffield Department of Population Health (J.C.H., C.M.v.D.), and Nuffield Department of Clinical Neurosciences (P.M.R.), University of Oxford, UK; National Institute of Neurological Disorders and Stroke (C.B.W.), NIH, Bethesda, MD; Institute of Cardiovascular and Medical Sciences, Faculty of Medicine (D.J.S.), and Robertson Centre for Biostatistics (I.F.), University of Glasgow, UK; German Center for Neurodegenerative Diseases (DZNE) (K.W., H.J.G.), Site Rostock, Greifswald, Germany; Interfaculty Institute for Genetics and Functional Genomics (U.V.), University of Greifswald, Germany; John Hunter Hospital (C.L.), Hunter Medical Research Institute and University of Newcastle, Callaghan, Australia; Neurovascular Research Group (NEUVAS) (J.J.-C.), Neurology Department, IMIM–Hospital del Mar, Barcelona, Spain; Institute of Cardiovascular Research (P.S.), Royal Holloway University of London & St Peters and Ashford Hospital, UK; Center for Human Genetic Research and Department of Neurology (J.R.), Program in Medical and Population Genetics, Broad Institute, Massachusetts General Hospital, Harvard Medical School, Boston; University of Cincinnati College of Medicine (D.W.), OH; Department of Neurology (J.W.C., S.J.K.), University of Maryland School of Medicine and Baltimore VAMC; Department of Neurology (J.F.M.), Mayo Clinic Jacksonville, FL; Department of Neurology (A.S.), Jagiellonian University, Krakow, Poland; Department of Clinical Sciences Lund, Neurology (A.L.), Lund University; Department of Neurology and Rehabilitation Medicine (A.L.), Skåne University Hospital; Department of Clinical Sciences Malmö (O.M.), Lund University, Sweden; Neuroscience Institute (R.P.G.), Saint Francis Medical Center, School of Health and Medical Sciences, Seton Hall University, South Orange, NJ; College of Public Health (D.K.A.), University of Kentucky, Lexington; Institute of Biomedicine (C.J.), the Sahlgrenska Academy at University of Gothenburg, Sweden; Department of Pharmacotherapy and Translational Research and Center for Pharmacogenomics, College of Pharmacy (J.A.J.), and Division of Cardiovascular Medicine, College of Medicine (J.A.J.), University of Florida, Gainesville; Department of Neurology (O.R.B.), University of British Columbia, Vancouver, Canada; Department of Epidemiology and Population Health (S.W.-S.), Albert Einstein College of Medicine, Bronx, NY; Stroke Center, Department of Neurology (J.-M.L.), Washington University School of Medicine, St. Louis, MO; Department of Medicine (B.D.M., P.F.M.), University of Maryland School of Medicine, Baltimore; Center for Public Health Genomics (S.S.R.), University of Virginia School of Medicine, Charlottesville; Durrer Center for Cardiovascular Research (F.W.A.), Netherlands Heart Institute, Utrecht, the Netherlands; Institute of Cardiovascular Science, Faculty of Population Health Sciences (F.W.A.), and Farr Institute of Health Informatics Research and Institute of Health Informatics (F.W.A.), University College London, UK; Peninsula Clinical School (V.S., C.M., M.C.), Frankston Hospital, Central Clinical School, and School of Clinical Sciences (T.P.), Monash Health, Monash University, Melbourne; Menzies Institute for Medical Research (V.S., R.M., M.C.), University of Tasmania, Hobart; Western Sydney University (R.T.), New South Wales, Australia; Departments of Neurology and Public Health Sciences (B.B.W.), University of Virginia, Charlottesville; Neuropsychiatric Institute (P.S.S.), Prince of Wales Hospital, Randwick, Australia; Munich Cluster for Systems Neurology (SyNergy) (M.D.), Munich, Germany; Intramural Research Program (L.J.L.), National Institute on Aging, NIH, Bethesda, MD; and University of Texas Health Sciences Center and Glenn Biggs Institute for Alzheimer's and Neurodegenerative Diseases (C.L.S., S. Seshadri), San Antonio.

## Glossary

1000G: 1000 Genomes reference panel
BI: brain infarcts
BMI: body mass index
BP: blood pressure
DBP: diastolic blood pressure
FLAIR: fluid-attenuated inversion recovery
GRS: genetic risk scores
GWAS: genome-wide association studies
HDL: high-density lipoprotein
IS: ischemic stroke
IS-SVD: small vessel disease subtype of ischemic stroke
IVW: inverse-variance weighting
L_10_BF: Log_10_ of Bayesian factor
LD: linkage disequilibrium
LDL: low-density lipoprotein
MAP: mean arterial pressure
PP: pulse pressure
SNP: single nucleotide polymorphism
SBP: systolic blood pressure
SSBI: small subcortical brain infarcts
SVD: small vessel disease
WMH: white matter hyperintensities
